# The diagnostic expertise acceleration module (DEAM): promoting the formation of organized knowledge

**DOI:** 10.1080/10872981.2019.1679945

**Published:** 2019-10-22

**Authors:** Brian Rissmiller, Danny Castro, Charles G. Minard, Moushumi Sur, Kevin Roy, Teri Turner, Satid Thammasitboon

**Affiliations:** a Department of Pediatric Critical Care Medicine, Baylor College of Medicine, TX, USA; b Dan L. Duncan Institute for Clinical and Translational Research, Baylor College of Medicine, TX; c Department of Academic General Pediatrics, Baylor College of Medicine, TX

**Keywords:** Deliberate practice, illness script, learning curve, diagnostic error, diagnostic expertise

## Abstract

**Background**: Ensuring that learners acquire diagnostic competence in a timely fashion is critical to providing high quality and safe patient care. Resident trainees typically gain experience by undertaking repetitive clinical encounters and receiving feedback from supervising faculty. By critically engaging with the diagnostic process, learners encapsulate medical knowledge into discrete memories that are able to be recollected and refined in subsequent clinical encounters. In the setting of exponentially increasing medical complexity and current duty hour limitations, the opportunities for successful practice in the clinical arena have become limited. Novel educational methods are needed to more efficiently bridge the gap from novice to expert diagnostician.

**Objective**: Using a conceptual framework which incorporates deliberate practice, script theory, and learning curves, we developed an educational module prototype to coach novice learners to formulate organized knowledge (i.e. a repertoire of illness scripts) in an accelerated fashion thereby simulating the ideal experiential learning in a clinical rotation.

**Design**: We developed the Diagnostic Expertise Acceleration Module (DEAM), a web-based module for learning illness scripts of diseases causing pediatric respiratory distress. For each case, the learner selects a diagnosis, receives structured feedback, and then creates an illness script with a subsequent expert script for comparison.

**Results**: We validated the DEAM with seven experts, seven experienced learners and five novice learners. The module data generated meaningful learning curves of diagnostic accuracy. Case performance analysis and self-reported feedback demonstrated that the module improved a learner’s ability to diagnose respiratory distress and create high-quality illness scripts.

**Conclusions**: The DEAM allowed novice learners to engage in deliberate practice to diagnose clinical problems without a clinical encounter. The module generated learning curves to visually assess progress towards expertise. Learners acquired organized knowledge through formulation of a comprehensive list of illness scripts.

## Background

How to bridge the chasm from novice to expert diagnostician *safely* and *efficiently* has always been the fundamental challenge of medical education. Traditionally, resident trainees have engaged in experiential learning by participating in repetitive clinical encounters with subsequent feedback from supervising faculty. Ideally, this experiential learning should allow the learner to engage critically in the diagnostic process and establish relationships that encapsulate medical knowledge into accessible memories within an individual’s mind. This form of organized knowledge with deep meaning is what distinguishes experts from novices[1]. In the setting of exponentially increasing medical complexity, a public outcry to minimize diagnostic errors while maintaining timely patient care[2], and current duty hour limitations, the opportunities to successfully practice diagnostic skills in the clinical setting (i.e. experiential learning) have become limited. For example, at our institution, in response to the duty hour changes and logistics related to clinical service complexity, our intern rotation in the ICU has lost ~25–33% of the time available for clinical learning. The lack of continuity of care created by shift work amplifies this problem by fragmenting the feedback loop inherent to the diagnostic process. This fragmentation creates a loss of opportunity which further limits the possibility of learning[2]. In order to bridge this chasm more efficiently, novel modes of learning must be implemented to augment traditional educational strategies.

A considerable amount of research in diagnostic thinking has shown that organization and availability of medical knowledge within clinicians’ memories are what make experts superior to novice diagnosticians [3,4]. Many models or constructs have been studied to understand how knowledge is organized and the interplay of this organized knowledge with a clinician’s diagnostic thinking [5–7]. The use of illness scripts as a framework to help leaners organize medical knowledge is among the most widely adopted approaches for successfully teaching diagnostic reasoning. [8–12]

We aimed to develop an educational module prototype to coach novice learners to formulate organized knowledge (i.e. a repertoire of illness scripts) in an accelerated fashion thereby simulating the ideal experiential learning in a clinical rotation.

## Methods

### Conceptual framework

**Figure 1.**
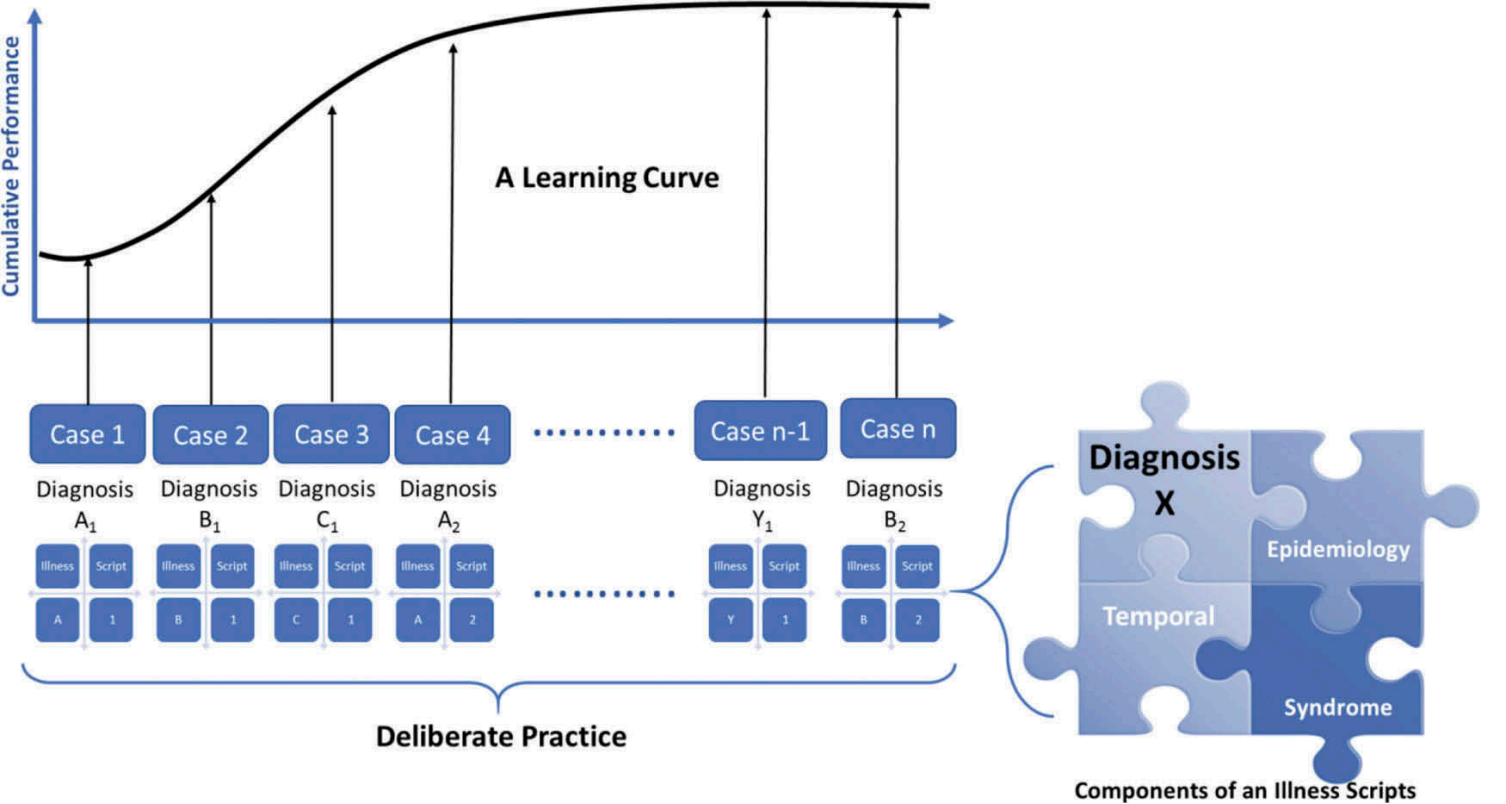
Conceptual Framework utilizing deliberate practice of diagnosing cases and creating illness scripts to accelerate learning as visually represented by a learning curve.

To represent the cognitive activity of organizing knowledge, we developed a conceptual framework (Figure 1) that incorporated deliberate practice, script theory, and learning curves to accelerate learning.

Successful deliberate practice is an effortful, repetitive training of key skills with immediate and informative feedback from an expert instructor[13]. Illness scripts are cognitive structures based on script theory which encapsulate organized and accessible clinical knowledge[14].An illness script contains three major components: epidemiology (age, underlying characteristics or health conditions); temporal relationship (onset, duration, progress), and syndrome (key signs/symptoms) [15,16]. Importantly, an illness script must be constructed by an individual learner and cannot be learned by memorization from an external source[17]. Learning curves are graphical relationships between effort (i.e. number of practice sessions) and learning achievement (e.g. score, pass rate, etc.) and can be used to quantify and analyze effort, rate, direction and maximal potential of learning[18]. These curves have been used to represent deliberate practice in clinical contexts[19].

### Module and case development

Using the proposed conceptual framework to guide the design, we developed the Diagnostic Expertise Acceleration Module (DEAM) prototype using respiratory distress cases in our Progressive Care Unit (PCU) as an exemplar. The DEAM is a computer-assisted, case-based learning module. The PCU is a 36-bed, intermediate care unit/step down ICU. The PCU is considered the most challenging rotation for our interns given the breadth and complexity of the patient population. In collaboration with a panel of five critical care experts, we identified the most common respiratory-based admission diagnoses to our PCU. We divided these diagnoses into broad categories which were initially based on a traditional ‘systems based’ approach of respiratory, cardiac, neurologic, toxic/metabolic, etc. Believing this system to be too simplistic and too similar to other educational approaches, we developed a 'physiology based' system that grouped illnesses based on primary respiratory signs. These major categories included wheezing, stridor, crackles, tachypnea/bradypnea with normal breath sounds and absent breath sounds. We believed this added layer of organization would be beneficial for the learners in establishing the patterns of the illness scripts and would more accurately mimic the level of expert thinking. Ultimately, we derived a consensus total of 35 illness scripts of which 29 were included in the final module (Table 1). For example, the expert illness script for laryngomalacia is *epidemiology*: infant, *temporal*: intermittent but recurrent, *syndrome*: inspiratory stridor, exacerbated by crying or agitation, improves with prone positioning.

**Table 1.** Diagnoses resulting in respiratory distress included as illness scripts in the DEAM. Number in parentheses indicates number of cases included in the final module.

Asthma (3)	Gastroesophageal reflux disease	Pyloric stenosis
Bronchiolitis (2)	Ingestion/narcosis (3)	Septic Shock (3)
Congenital Heart Disease (2)	Laryngomalacia (2)	Seizure (2)
Croup (2)	Atelectasis (2)	Tracheoesophageal fistula
Diaphragm Paralysis	Myocarditis (2)	Tracheostomy displacement (2)
Diabetic Ketoacidosis	Pain	Tracheostomy obstruction
Empyema	Pancreatitis	Tracheitis (2)
Foreign Body	Pulmonary Embolism (2)	Tracheomalacia
Fever (2)	Pneumonia (3)	Vocal cord dysfunction (2)
Guillain-Barre syndrome	Pneumothorax (2)	

A Monte Carlo simulation was used to estimate the number of cases required to demonstrate an acceptable level of performance using Cumulative Summation methods[20]. About 90% of 10,000 simulated trials reached an acceptable level of performance within 50 cases assuming an acceptable failure rate ≤5%, an unacceptable failure rate ≥30%, 10% decreased odds of failure per case, and 10% type 1 and 2 error rates. While there are no established standards for these statistical measures, these assumptions target an educational intervention wherein the average learner achieves a correct answer 70–95% of the time and conservatively learns by ~10% with each question. The expert panel then authored 50 cases for the module, including a short history, physical exam and diagnostic data, and specifically designed each case to include the essential aspects of the illness scripts. The panel’s intent was for each case to have equivalent difficulty.

### Initial module revision

The module was initially tested by four individuals (2 attendings, 1 fellow, 1 nurse practitioner) using Select Survey® software. This initial test resulted in three major revisions to the final module. First, the module was transitioned to an external website with a Moodle® based software platform to allow learners to access the module from any computer. Second, there were 15 to 17 possible answer choices from which the learner initially chose the correct diagnosis. This was reduced to 10 in the final module to eliminate redundancy and reduce distraction – for example heart failure, myocarditis, and congenital heart disease were no longer all present as possible answer choices. The third improvement was to refine the illness scripts in order to eliminate ambiguity – for example, community-acquired pneumonia was changed to pneumonia and the scripts for vascular ring (too rare an entity and lacking distinctive history or physical exam findings) and cystic fibrosis (too specific to be diagnosed within case) were removed.

### Validation with experts, experienced and novice learners

**Figure 2.**
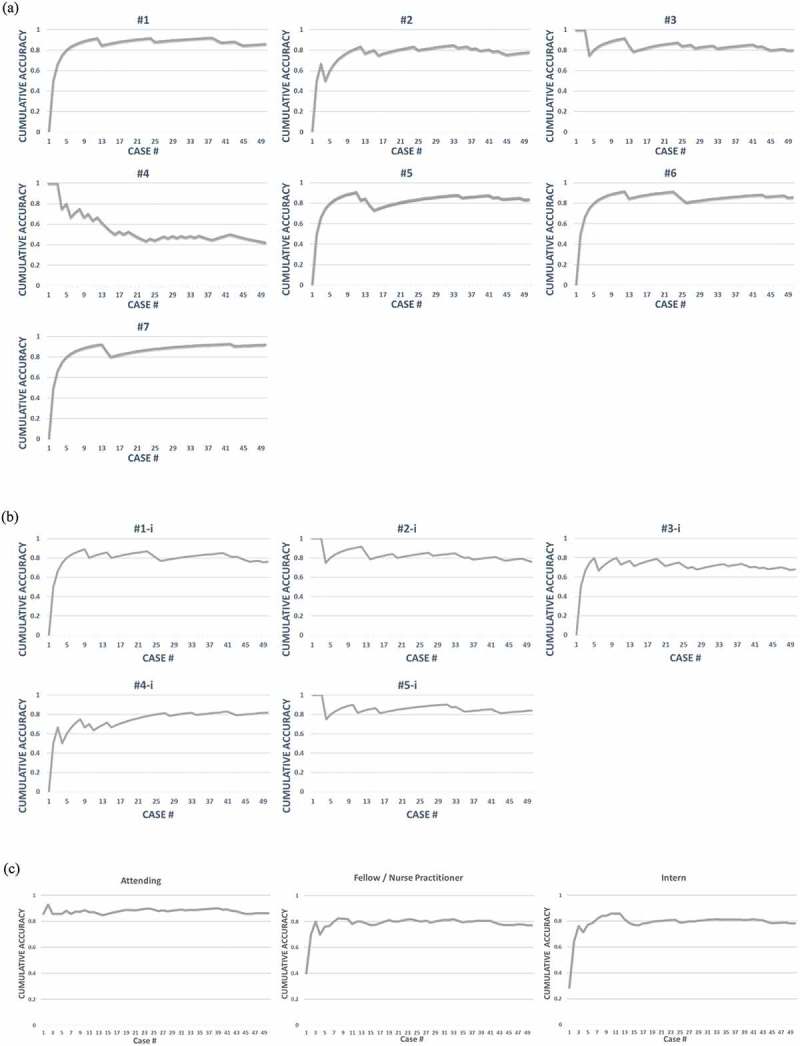
DEAM Learning Curves. For each case, the learner reads the case and selects a diagnosis. The module calculates a cumulative accuracy (percent correct) after each case. At the conclusion of all 50 cases, the module plots the individual’s learning curve as well as a group curve. a) Individual curves for the 7 Fellows/Nurse Practitioners, b) Individual curves for the 5 Interns who completed all 50 cases, c) collective group curves distributed by level of training.

In order to assess our module prototype, we sought to query the continuum of experience – namely, experts, experienced learners and novices. The experts were critical care attendings and the experienced learners were critical care fellows and nurse practitioners. The novices were pediatric interns at the start of their residency. Both groups of learners performed the same four steps for the deliberate practice of making a diagnosis and formulating an illness script. Each participant 1) read a scenario and diagnosed the disease process from a selection of answers, 2) received the correct answer without further feedback or explanation, 3) created an illness script and 4) received a consensus expert illness script for comparison (See Appendix A, example case). Using the answers from the first step, cumulative percent accuracy was plotted over the number of cases studied to generate individual and group learning curves (Figure 2) for feedback on the individual’s progressive learning and performance. The Institutional Review Board of Baylor College of Medicine approved this project (H-35103).

We evaluated how the various experts and experienced learners interacted with the module. In this way, we could verify the content of the cases while testing the ability of the module to collect data that generated learning curves. Using a survey as well as module analytics, we assessed the feasibility of the user interface, specifically the time to complete the module and each learner’s evaluation of the module.

Next, we piloted the module with the incoming interns in May/June 2016. The participants received an email introducing the study which included a 1-minute video introducing the module interface and the basics of an illness script as a package of organized knowledge which included semantic qualifiers in three domains (epidemiology, temporal relationship, and syndrome). Individuals were allowed access to the DEAM for 30 days. Each individual participant was required to work on the cases in the prescribed order (1–50), but could complete the cases at their own pace. We collected the same analytic data for comparison with the experts and experienced learners.

## Results

A total of seven expert attendings, and seven experienced participants including four nurse practitioners with more than 2 years of critical care experience, and three critical care fellows (one in the first year and two in the second year of training) completed the module. From the 50 interns invited to participate in the pilot, 19 started and 5 completed all 50 cases within the module. An additional 5 completed more than 8 cases, and the remaining 9 completed 5 or fewer cases.

### Learning curve generation

The DEAM pilot data demonstrated meaningful learning curves on diagnostic accuracy for individual learners as well as groups of learners (Figure 2). Considering all the participants, the expert attending data means generated a group curve that reached a sustained plateau at approximately 90% accuracy, representing the maximal learning potential for the module. The fellow/nurse practitioner curve as well as the intern curve reached a plateau at ~80% (Figure 2(c)). For the 5 interns who completed all 50 cases, the DEAM yielded learning curves which were similar in shape to the experienced learner’s curve (Figure 2(a,b)). All of the learning curves reached a plateau within about 14 cases, which we believe is indicative of rapid acclimation to the testing environment. There was one individual in both the experienced learners (Figure 2(a), #4) and novice (Figure 2(b), #3-i) groups whose sustained plateau did not approach the maximal learning potential for their level of experience. For the 3 novice learners that completed at least 14 cases but not the entire module, one individual answered the first 13 cases correctly before reaching a plateau of ~85% accuracy over 24 cases. A second individual’s curve was similar to Figure 2(b), #5-i with a plateau at ~75% over 19 cases. The final individual’s curve was similar is shape to Figure 2(b), #4-i and was approaching 80% over 14 cases.

### Time to complete module

For the experts and experienced learners, the median time spent per case was 2.3 min (Interquartile range-IQR: 1.4–4.2), or approximately 3 cumulative hours to complete the entire module over multiple sittings. The median time the novices spent per case was slightly longer 2.5 min (IQR: 1.9–4.3), and the average cumulative time to complete all cases was comparable at 2.8 h.

### Learner evaluation

Overall, feedback for the module was positive. On a 5-point Likert scale (1 – strongly disagree to 5 – strongly agree), the learners rated the module (mean ± SD) at 3.9 ± 0.5 for improving the ability to diagnose a cause for respiratory distress and at 3.8 ± 0.9 for improving the ability to create an illness script. The experts and experienced participants commented that they were challenged to ‘think critically’ and that ‘practice of making illness scripts is a good one, forces you to take [making] diagnosis to another level.’ No novice reported dissatisfaction with the time to complete the module. All of the comments from the novices are reported below:

‘Nice variety. Practice of making illness script is a good one’

‘I should be able to make [diagnosis] faster and make the correct diagnosis with more [confidence].’

‘Would be helpful to choose choices for illness scripts as an example of what is expected.’

“Really enjoyed this set of modules. Highly recommend before starting a [PICU] rotation. “

### Case performance analysis

When analyzing the learners’ performance on diagnoses with multiple cases within the module, we identified five representative patterns. Table 2 summarizes these five diagnoses, including case numbers within the module and mean expert and novice score. The first pattern is demonstrated by the asthma cases which had a relatively high but flat performance, ideally indicative of deliberate practice of an appropriately difficult case. The bronchiolitis cases were universally answered correctly by both expert and novice learners. While this would be expected from even novice pediatricians, this pattern may also indicate that these cases are too easy. A subset of cases, such as fever manifesting as acute respiratory distress, had universally poor performance which likely indicates an unclear question or diagnosis. The myocarditis cases demonstrated an optimal pattern for successful deliberate practice. There was improvement from the first to the second case for novice learners as well as high performance by experts and experienced learners. Finally, the pneumonia cases demonstrated an unexpected decline in performance with each subsequent case. This pattern indicates a set of cases that may be unclear (similar to the fever cases) or that contain confounding information in the latter case. For example, the third question in this set included a patient with a tracheostomy which may have contributed to the decline in learner performance.

**Table 2.** Mean accuracy scores for representative diagnoses and cases (see Appendix B for all cases). Bold scores are less than 0.70.

		Expert/experienced providers			Novices		
Diagnosis:	Case #	MEAN SCORE			MEAN SCORE		
Asthma	12, 20, 27	0.86	0.86	1.00	1.00	**0.60**	1.00
Bronchiolitis	2, 17	1.00	1.00		1.00	1.00	
Fever	1, 49	**0.57**	**0.64**		**0.40**	**0.40**	
Myocarditis	4, 47	0.71	0.93		**0.40**	1.00	
Pneumonia	3, 26, 28	0.86	0.71	0.71	1.00	**0.60**	**0.40**

### Illness script analysis

After reviewing the illness scripts that were entered by the learners in the DEAM, the vast majority of both experienced and novice learners completed the free text response illness script questions for all 50 of the cases. We generally observed that for the cases which repeated diagnoses that the quality of the illness script improved with each attempt. For each of the diagnoses listed in Table 2, Table 3 enumerates the illness scripts which were entered by a single, representative novice (Table 3) and expert (Table 3). The table includes the diagnosis, whether the learner correctly or incorrectly answered the question, and then the full and unedited illness scripts – including epidemiology (E), temporal relationship (T), and syndrome (S) – that were entered by the representative user. For each set of cases, there was a progressive improvement in the quality of the illness script developed by both the novice and expert learners. Our commentary of this improvement is summarized in the final column of Table 3.

**Table 3a.** Representative *Novice* learner illness scripts as entered by learner and author commentary. Bold text indicates that diagnosis was incorrect, E = epidemiology, T: temporal relationship, S: syndrome.

Diagnosis		Illness script 1st case attempt	Illness script 2nd case attempt	Illness script 3rd case attempt	Commentary
Asthma		E: Children > 2 yrs T: Intermittent, worse with environment, exercise, allergens, other triggers S: Cough, chest tightness, difficulty breathing, shortness of breath, wheezes	E: Children > 2 yrs T: Chronic cough with worsening exacerbations, worse at night S: Polyphonic cough, expiratory wheezes, no signs of infection, signs of atopy, family history, history of trigger factors	E: Children > 2 yrs (preschool, adolescent) T: Chronic with intermittent acute exacerbations S: Respiratory distress, difficulty breathing during acute attacks, polyphonic bilateral wheezes, no signs of infection on CXR, diminished breath sounds	Refined all 3 components to include pertinent, significant details with each question
Bronchiolitis		E: < 2 years old, most often from 2–6 months T: symptoms peak on days 5–7, typically resolves in 2–3 weeks S: cough, congestion, tachypnea, hypoxemia, wheezing, fever	E: < age 2, typically 2–6 months T: ~10 day clinical course, with peak symptoms days 5–7 S: cough, congestion, URI symptoms followed by wheezing, retractions, resp distress		Refined Temporal (included diagnostic information) and Syndrome (pathognomonic details)
Fever		**E: Post-op fever** **T: Days 1–5 after surgery** **S: Unexplained fever after surgery**	E: febrile illness T: acute S: fever, tachycardia to 10 bpm per 1.8 degrees		Included details from expert script in 2^nd^ question
Myocarditis		**E: All ages** **T: After viral-like illness** **S: Gallop, fever, weakness, hypotension, distended neck veins, hepatomegaly, lower extremity edema**	E: Any age T: Acute symptoms, often following viral prodrome S: Gallop, enlarged cardiothymic silhouette, signs of heart failure (bilateral lung infiltrates, hepatomegaly), fever, change in mental status, gallop		Increased details in Syndrome in 2^nd^ script, high quality script even though incorrect answer
Pneumonia		E: Any age group T: 5–7 days S: Lobe infiltrates	E: Any age T: Sub acute S: Fatigue, cough, crackles diffuse	**E: Any age** **T: Subacute progressive** **S: Fever, crackles, opacity on CXR**	Richer details in each iteration (more specific semantic qualifiers)

**Table 3b.** Representative *Expert and Experienced* learner illness scripts as entered by learner and author commentary. Bold text indicates that diagnosis was incorrect, E = epidemiology, T: temporal relationship, S: syndrome.

Diagnosis		Illness script 1st case attempt	Illness script 2nd case attempt	Illness script 3rd case attempt	Commentary
Asthma	**E: Children, more common with atopy, history of asthma in parent** **T: chronic condition with episodic exacerbations** **S: shortness of breath, chest pain, chest tightness, worse with activity, cough at night, prolonged expiration, wheezing, and decreased breath sounds on exam**		E: Children of all ages, more common > age 5; T: chronic condition with intermittent exacerbations S: chest pain, SOB, cough, wheeze, worse at night or with URI	E: school aged children T: chronic illness with intermittent exacerbations S: cough, wheeze, intermittent exacerbations of symptoms associated with URI, + family history atopy, eczema	Refined script by transitioning information from Epidemiology to Syndrome
Bronchiolitis	E: (n/a) T: symptoms occur after viral infection (mostly RSV) and usually occurs 3–5 days after getting infected S: fever, cough and dyspnea with wheezing without bronchodilator response		E: infant and children less than 2 years T: shortly 2–3 days after viral infection (most likely RSV) S: Fever, cough, rhinorrhea and wheezing. Chest x-ray shows hyper expansion and focal atelectasis		Included CXR details from expert script and added response for Epidemiology
Fever	**E: children, viral vs bacterial, can be post-operative** **T: common in first 24 hours post-op** **S: atelectasis/pneumonia, viral or bacterial infection, wound infection, DVT, drug reaction**		E: infants and children T: fever S: tachypnea with improves/resolves with defervescence, non-focal pulmonary exam		Focused to key qualifiers in 2^nd^ script
Myocarditis	E: any age group T: can be post-viral; any time of year S: crackles B/L, diffuse infiltrates, liver edge, gallop, SOB		E: any age; setting of viral illness T: acute S: gallop, crackles, CXR with cardiomegaly, liver edge down, in setting of viral illness		Included addition detail of CXR in 2^nd^ script
Pneumonia	E: 7yo, sick contacts T: acute S: fever, abdominal pain, low SpO2		E: sick contacts T: acute S: febrile, + CXR	**E: trach/vent dependent** **T: acute** **S: secretions, desaturation**	Limited to crucial details, seemed to be ‘distracted’ by presence of tracheostomy in 3^rd^ question

## Discussion

Conceptual frameworks grounded in educational theories and best practices can guide the development and evaluation of an educational innovation. This study describes a learning module that uses deliberate practice principles to teach learners to formulate an illness script, a surrogate for organized knowledge, with a quantitative outcome in learning curves. Our proposed approach appears to be a feasible and effective method for learners to create and refine a variety of illness scripts even in the absence of a clinical encounter. The clinical reasoning literature purports that elaborated illness scripts are the end products of years of clinical learning experience [8,21] and, therefore, teaching learners about illness scripts by other means is difficult to impossible[17]. Our preliminary evidence – that the novice interns in our study created increasingly robust scripts as they progressed through the module – challenges this notion by expanding the definition of experiential clinical practice.

Our study adds another chapter to the utility of learning curves in medical education. Pusic has published extensively on the use of learning curves in the interpretation of radiographs by emergency medicine physicians as well as the generation and analysis of learning curves in general [18,22,23]. Similar to Pusic’s results, the learning curves in our study provide valuable information regarding a learner’s initial and peak performance. Most importantly, they can quickly and effectively identify the struggling learner and thus allow for timely assessment and intervention. This capability opens the possibility of using our module as an on-boarding tool to accelerate learning for residents prior to a scheduled rotation. With the proper content, a DEAM could assess a novice learner on a standard set of cases (content knowledge) and assess the ability to formulate an illness script (knowledge organization). By being able to compare an individual to their current peers (or a historical data set), the learning curve could provide the learner with motivation to improve during their rotation. The educator could also gain insight into which learners may need extra preparation or remediation upon starting a rotation. Additionally, our data demonstrate that even experienced practitioners (Figure 1(a), #4) may have deficits and highlights the potential utility of our module as a tool not only for on-boarding novices but also for ensuring competency of experienced providers in a form of continuing education. Learner #4 did complete the free text questions for every illness script with appropriate and case-specific information which decreases the likelihood of random guessing as an explanation for below average performance. We would expect the learning curve for someone who was randomly selecting answers to never reach a plateau level which would inform the educator to investigate the learner’s ability or willingness to engage with the module.

As with any educational innovation, the content of what we are trying to teach impacts both the perceived and actual learning. Cases that seemed obvious to the authors may be misinterpreted by the learners (e.g. the fever cases). Our proof of concept study offers encouraging evidence that cases can be designed with the proper balance of desirable difficulty and deliberate practice (e.g. myocarditis and asthma cases) to challenge learners to encode the knowledge without leading to frustration and abandonment of the module. We are encouraged that those who completed our entire module described an appropriate balance in this regard. Given the low response rate from our interns, we surmise that the time we offered the module, just prior to starting residency, may not be practical. In future studies, we plan to assess the optimal timing for this DEAM.

This is the first attempt to teach the challenging concept of illness script formation via an online module, and our preliminary data indicate that our DEAM allows for successful deliberate practice of the creation of an illness script. Further work remains to understand how to best assess the quality of illness scripts created and provide impactful feedback to each learner about their illness scripts.

During this pilot study, the process of creating the conceptual framework, cases and the DEAM taught us many lessons. Table 4 contains a summary of lessons learned regarding the use of deliberate practice, for teaching illness scripts, and for utilizing computer-assisted modules in graduate medical education.

**Table 4.** Tips and lessons learned.

Tips for using deliberate practice in graduate medical education:	Tips for teaching illness scripts in graduate medical education:	Tips for using computer-assisted modules in graduate medical education:
Feedback should be immediate and specific Balance difficulty of deliberate practice with expected endurance of learners (i.e. too many cases will be discouraging and lead to drop out/disinterest) Deliberate practice can be difficult, ensure that this difficulty is desirable and not overly frustrating Utilize a variety of question types (e.g. multiple choice and short answer) to increase retention and encoding of information	Orient the learner to the concept of illness scripts Organize knowledge so that it can be easily understood, replicated, and applied Consider the experience level of learners when developing the content- true novices will need to learn the cases as well as practice creating illness scripts, while more experienced learners will likely only be practicing (and improving) their repertoire of scripts Deconstruct learning experiences into subsets of cases instead of giving them all at one time	Avoid redundant steps. Today’s learners do not need deliberate practice of clicking buttons Consider both time per individual case as well as total time for the entire module Optimize efficiency of the user interface as it can impact learner performance Design a simple module. The more complex the module, the more likely something is to break Ascertain software compatibility for ease of access to the module (i.e. address firewall issues)

This study has some limitations. We assumed that learning from a written case presentation can mimic an experiential learning from a clinical encounter. It remains to be seen whether utilizing a case presentation to prompt a learner to formulate his or her script can approximate the cognitive effort required to create and encode an illness script. Since this was an asynchronous online learning, we did not have control over potential contaminations from learning within participants or from others outside the study. Our pilot study was neither designed nor powered to interrogate these possibilities.

Through an iterative process, we attempted to create cases that would provide enough difficulty to distinguish performance between novice and experienced learners. Since the learning curves for our novice and experienced learners were similar, our set of pilot cases may not be able to differentiate between these groups. We will need to continue to assess the balance between the difficulty of the cases and our learner’s level of experience to determine if we can (and should) distinguish these sub-groups. Though the DEAM is presented as a series of test questions, it is supposed to serve as an assessment for learning.

Finally, our DEAM presented a case set in a predefined order for all learners. While this allowed us to compare cases with the same diagnosis, it limits our ability to analyze the cases completely as we are unable to determine if learner performance is solely dependent on the case itself or on the case sequence. Additionally, as can be seen in Appendix B, approximately equal number of diagnoses had a decrease in performance between cases as had an increase. Given the small sample size in this pilot phase, we cannot conclude whether this finding was due to suboptimal learning from the illness script, content of our cases and confounding information in the case stems. It is possible that more repetition of a case (i.e. deliberate practice) is required to ascertain a stable diagnostic performance. As more learners utilize our DEAM in the future phase, we will be able to adjust both the case content and sequence to ensure optimal learning.

### Future/next steps

We plan to continue to refine this prototype with a larger population of learners in order to generalize the findings from the pilot study. As more data is collected, we will be able to gain deeper insight into the information provided by learning curves as well as enhance our ability to understand how our content affects the learning process.

We aim to create a comprehensive educational program using a DEAM platform to allow accelerated learning prior to or early in a clinical rotation. In this way, residents will be primed with a core set of illness scripts which they can further recalibrate and optimize during their clinical rotation with actual patient encounters. We, also, hope to optimize the feedback on the illness scripts generated by the learner in order to enhance the retention of acquired knowledge. Finally, we hope to develop a post-test to determine if the learner has achieved and retained mastery of basic principles required to create an illness script.

## Appendix A: Representative case in DEAM utilizing 4 steps to emphasize deliberate practice

**Step 1)**

**Step 2)**

**Table:**

**The learner reads a scenario and diagnoses the disease process from a drop down menu**
HPI: 8 month old female with cleft palate presents to the PCU for post op care following repair of her soft palate. She does well on POD #0, but on examination the next morning, she is fussy and refusing to eat. The bedside nurse reports that the surgical team evaluated the palate early this morning and everything was perfect. The patient has had good urine output overnight Temp: 101.5 RR: 50 HR: 135 BP: 90/48 SaO2: 99% on room air Gen: awake and alert, crying until held by parent HEENT: extra-ocular movements intact, tympanic membranes normal, mucous membranes moist Resp: clear to auscultation bilaterally, no wheezing, no retractions CV: normal S1/S2, regular rhythm, I/VI systolic murmur Abd: soft, non-distended, non-tender, liver edge at costal margin Ext: warm, cap refill < 2 seconds Skin: (+) cracked dry skin over diaper area Labs: none; CXR: none
Answer choices in menu: asthma, bronchiolitis, diaphragm paralysis, fever, myocarditis, ingestion, pain, pneumonia, pneumothorax, seizure

**Table:**

**The learner receives feedback with the correct answer without further explanation**
Example correct answer to case in Step 1: fever

**Step 3)**

**Table:**

**The learner creates an illness script**
Example answer entered by learner: **Epidemiology:** any age **Temporal**: coincides with elevated temperature **Syndrome**: increase in RR according to temperature

**Step 4)**

**Table:**

**The learner receives a consensus expert illness script for comparison and self-education**
Expert illness script: **Epidemiology:** any age group **Temporal**: symptoms concurrent with fever **Syndrome**: Tachypnea 5–10 breaths per 1.8 °F, normal BS, tachypnea resolves with fever control

## Appendix B: DEAM Cases and Scores: Bold scores are less than 0.70

**Table:**

		EXPERT/EXPERIENCED PROVIDERS			NOVICES		
Diagnosis:	Case #	MEAN SCORE			MEAN SCORE		
Asthma	12, 20, 27	0.86	0.86	1.00	1.00	**0.60**	1.00
Bronchiolitis	2, 17	1.00	1.00	.	1.00	1.00	.
Congestive heart failure in CHD	21, 33	0.86	0.93	.	0.80	1.00	.
Croup	29, 40	1.00	**0.64**	.	1.00	0.80	.
Diaphragm paralysis	15	0.79	.	.	**0.60**	.	.
Diabetic Ketoacidosis	23	0.93	.	.	1.00	.	.
Empyema	19	1.00	.	.	1.00	.	.
Foreign Body	36	0.93	.	.	1.00	.	.
Fever	1, 49	**0.57**	**0.64**	.	**0.40**	**0.40**	.
Guillain-Barre syndrome	5	0.93	.	.	1.00	.	.
Gastroesophageal Reflux Disease	8	1.00	.	.	1.00	.	.
Ingestion/narcosis	22, 32, 35	0.93	0.79	0.93	1.00	0.80	**0.40**
Laryngomalacia	6, 25	0.93	**0.50**	.	0.80	**0.60**	.
Atelectasis	14, 39	**0.57**	0.86	.	**0.60**	0.80	.
Myocarditis	4, 47	0.71	0.93	.	**0.40**	1.00	.
Pain	30	0.93	.	.	1.00	.	.
Pancreatitis	43	0.71	.	.	**0.40**	.	.
Pulmonary Embolism	34, 45	0.79	**0.43**	.	**0.40**	0.80	.
Pneumonia	3, 26, 28	0.86	0.71	0.71	1.00	**0.60**	**0.40**
Pneumothorax	31, 38	1.00	0.93	.	1.00	1.00	.
Pyloric stenosis	41	1.00	.	.	0.80	.	.
Septic shock	9, 18, 48	0.86	0.91	0.93	0.80	1.00	0.80
Seizure	37, 50	0.86	0.79	.	0.80	0.80	.
Tracheoesophageal Fistula	13	**0.50**	.	.	0.80	.	.
Tracheostomy displacement	24, 44	0.93	**0.29**	.	0.80	**0.40**	.
Tracheostomy obstruction	10	1.00	.	.	0.80	.	.
Tracheitis	16, 42	0.86	**0.50**	.	0.80	**0.20**	.
Tracheomalacia	7	0.86	.	.	1.00	.	.
Vocal Cord dysfunction	11, 46	0.79	0.86	.	**0.40**	0.80	.
